# COVID Stress and Mental Health Among Sexually Diverse Couples

**DOI:** 10.1007/s13178-025-01125-4

**Published:** 2025-06-24

**Authors:** Madison Shea Smith, Michael E. Newcomb

**Affiliations:** Institute for Sexual and Gender Minority Health and Wellbeing, Northwestern University, Chicago, IL USA

**Keywords:** COVID, Mental health, Couples, Sexual minority, Heterosexual

## Abstract

**Introduction:**

It is now well-established that the COVID- 19 pandemic had profound mental health impacts that were not distributed equally throughout all portions of the US population. In this study, we used mental health and COVID stress data from the National Couples’ Health and Time (NCHAT) study to test the differential impact of COVID stress on key indicators of mental health (e.g., depression, anxiety) among sexually diverse couples.

**Methods:**

We leveraged a sample of *N* = 1515 couples (3030 individuals) from the NCHAT study, who filled out self-report measures of COVID stress (e.g., concerns about acquiring COVID, immunocompromised household members), depression, anxiety, general stress, emotion regulation, and positive/negative coping behaviors in 2020 and 2021. These data were submitted to a series of constrained latent variable actor-partner interdependence models.

**Results:**

COVID stress had widespread impacts on mental health problems for groups identifying as gay/lesbian, straight, and plurisexual. However, people identifying as plurisexual (e.g., bisexual, pansexual) and their partners tended to experience: (1) a stronger COVID stress-anxiety association compared to people identifying as gay/lesbian and straight and (2) tended to be more dissimilar with their partners on mental health.

**Conclusions:**

These results imply unique impacts of COVID stress among subgroups of people identifying as plurisexual and their partners, as well as prevention approaches for further study.

**Policy Implications:**

Our study suggests that reliance on one’s romantic partner may be key to incorporate in future public health messaging yet may not be as beneficially impactful for all groups (e.g., people identifying as plurisexuals).

**Supplementary Information:**

The online version contains supplementary material available at 10.1007/s13178-025-01125-4.

Although the COVID- 19 pandemic^1^ prompted mental health concerns among most portions of the US population, people identifying as sexual minorities (SMs) endorsed more COVID-related stressors and mental health problems in response to the emergence and continuance of COVID- 19 (Abramovich et al., 2022; Bavinton et al., 2022; Bécares & Kneale, 2022). In the present study, we leverage a nationally representative dataset to examine how COVID stress may have differentially impacted SM individuals’, and their partners’, mental health. Although the early days of the COVID- 19 pandemic are now past, it is important to interrogate such large, broadly impactful events in light of U.S. population health, and particularly for minoritized portions of the population. Doing so can shed light on health broadly and suggest ways to support public health in future scenarios.

## Disparities in Health for Sexual Minorities During COVID- 19

Mental health disparities are well-documented in people identifying as SMs relative to those identifying as heterosexuals (Cochran & Mays, 2006; Meyer, 2003; Wittgens et al., 2022). These disparities were worsened during the COVID- 19 pandemic, and national studies of people identifying as sexual and gender minorities (SGMs) find that these individuals tended to experience worse outcomes across nearly all measures of well-being (Gutman et al., 2022). For instance, people identifying as SGMs experienced higher rates of depression, anxiety, posttraumatic stress, and substance use during COVID- 19 compared to people identifying as heterosexual and cisgender (Bavinton et al., 2022; Bécares & Kneale, 2022; Bleckmann et al., 2022; Hart et al., 2022; Herrmann et al., 2022; Jacmin-Park et al., 2022; Leyva-Moral et al., 2022; Mullin et al., 2022; Oren, 2022; Pease et al., 2022; Wang et al., 2022), and compelling evidence now suggests that these worsening disparities are attributable to higher rates of COVID stress, which people identifying as SGMs also endorsed more frequently and intensely than people identifying as heterosexual (Abramovich et al., 2022; Bavinton et al., 2022; Bleckmann et al., 2022; Goldey et al., 2022; Oren, 2022). Indeed, these COVID-specific stressors (e.g., stay at home orders, concerns about acquiring COVID) seem to have compounded existing structures of inequity to worsen mental health for people identifying as SGMs (Abramovich et al., 2022; Bavinton et al., 2022; Bécares & Kneale, 2022; Bleckmann et al., 2022; Dyar et al., 2022; Fallahi et al., 2022; Gutman et al., 2022; Herrmann et al., 2022; Oren, 2022; Pease et al., 2022; Smout et al., 2022; Timmins et al., 2022; Wang et al., 2022). These existing structures are well-described by theories of minority stress, which state that the discrimination, victimization, and other stigmas perpetuated at multiple levels (e.g., societal, interpersonal, intrapersonal) lead people identifying as SMs to be disproportionately vulnerable to a variety of negative health outcomes, including mental health (Brooks, 1981; Diamond & Alley, 2022; Hatzenbuehler, 2009; Meyer, 2003).

Because COVID stress impacted the entire US population—but not at equal rates—studying its differential impacts on portions of the population at greater risk was an overarching aim of this study. This was investigated using couples’ data, since romantic partnerships are a key source of resilience for all individuals, but particularly for SMs, in buffering against poor mental health (Feinstein et al., 2016a, 2016b; Newcomb, 2020; Sarno et al., 2021; Simon & Barrett, 2010; Whitton et al., 2018). In addition, couples’ data provides the unique opportunity to understand how each partners’ reports of a phenomenon—in this case the early COVID- 19 pandemic—can enter into their relationship to impact the wellbeing of both partners. This is a key innovation, especially because romantic partners were often individuals’ sole source of in-person support during COVID- 19. Understanding these crossover effects within couples (between partners) is not possible using data collected solely from individuals (e.g., where individuals in relationships report on their partners’ characteristics).

## Impacts of COVID- 19 for SM Couples

Although SM couples are exceedingly similar to non-SM couples (Kurdek, 2004), they also inhabit a unique sociocultural environment, which the COVID- 19 pandemic may have differentially affected. That is, the uncertainty wrought by the COVID pandemic may have been compounded for SMs, who are also exposed to unreliable societal safety nets (e.g., acceptance from family, friends, peers; Diamond & Alley, 2022) and structural oppression (Brooks, 1981; Meyer, 2003). This may have created a particularly unstable socioemotional environment for SM couples (Bleckmann et al., 2022; Salerno & Boekeloo, 2022; Salerno et al., 2022; Westwood et al., 2022; Zhang et al., 2022), leading to a breakdown of usual coping (Houghtaling et al., 2024), and in turn worsening mental health (Diamond & Alley, 2022; Fallahi et al., 2022; Smout et al., 2022; Timmins et al., 2022; Tüzün et al., 2022). The same would not be true of couples in which both partners identify as heterosexual and cisgender.

It is also possible COVID stress impacted SM and non-SM couples similarly. For instance, SM relationships function similarly to non-SM ones in terms of satisfaction, longevity, and commitment (Kurdek, 1992, 1994, 2004; Kurdek & Schmitt, 1987), and people identifying as SMs are usually resilient to minority stressors (Bariola et al., 2015; Toze et al., 2022), in part through the support of romantic partners (Sarno et al., 2021). When COVID restrictions (e.g., stay-at-home orders) “forced” even people who do not identify as SMs to rely on cohabiting partners as a sole source of support, this may have temporarily leveled the playing field between people identifying as SMs and people identifying as non-SMs in types of coping (Houghtaling et al., 2024), meaning that sequelae of COVID stress would be similar in SM and non-SM couples.

## Current Study

Prior studies using the current dataset have documented disparities in COVID stress between partnered people identifying as SMs and those identifying as heterosexual (Kamp Dush et al., 2022; Manning & Kamp Dush, 2022), as well as differences in rates of positive and negative coping (Houghtaling et al., 2024). However, no research to our knowledge has investigated the impact of COVID stress on mental health within dyads. In the present report, we leverage couples’ data to test complex interrelations between COVID stress and mental health, separately for people identifying as SMs and people identifying as non-SMs. In doing so, we chose to separate people identifying as plurisexual from people identifying as monosexual (i.e., gay/lesbian), because prior research has shown that relationship involvement may be iatrogenic among people identifying as plurisexual (Feinstein et al., 2016a, 2016b). In part, this is because people identifying as plurisexual face unique minority stressors because of their relationship status (e.g., assumptions of sexual orientation/identity) or from romantic partners themselves (e.g., suspicions—based in stereotypes—about one’s ability to remain monogamous; Feinstein et al., 2016a, 2016b; Ochs, 1996). We also include diverse outcomes on a spectrum from behavior (e.g., coping) to mental health syndromes (e.g., depression) to fully elucidate our associations of interest. In all, our goal was to examine whether, to what extent, and among whom COVID stress had a disproportionate impact on mental health.

## Methods

### Participants

Participants were 1515 couples (*N* = 3030 individuals) from across the United States enrolled in the NCHAT study (Kamp Dush & Manning, 2023). Participants were, on average, 42.39 years old (SD = 10.81). In terms of sexual orientation/identity, 782 participants (25.8%) identified as gay/lesbian, 1767 (58.3%) identified as straight, 412 (13.6%) identified as plurisexual, and 69 (2.3%) did not self-identify. In terms of gender identity, 1438 (47.5%) identified as cisgender men, 1390 (45.9%) identified as cisgender women, 48 (1.6%) identified as transgender, and 97 (3.2%) identified as nonbinary or some other gender (e.g., agender, genderqueer).^2^ Participants’ modal level of education was a bachelor’s degree (*N* = 891, 29.4%), and on average, participants reported a yearly household income of $123,214.80 (SD = $108,181.40).^3^ Race/ethnicity data is listed in Table 1.

**Table 1:** Race and ethnicity of individual NCHAT respondents (*N* = 3030)

	White	Black or African American	American Indian or Alaska Native	Asian Indian	Chinese	Filipino/a/x	Japanese	Korean	Vietnamese	Native Hawaiian/Pacific Islander	Other Asian	Some other race
*N*	2396	327	102	53	76	41	27	36	23	21	30	155
%	79	11	3	2	3	1	1	1	1	1	1	5
Are you Hispanic, Latino/a/x, or Spanish or Spanish origin?												
	No	Yes, Mexican, Mexican Am., Chicano/a/x	Yes, Puerto Rican	Yes, Cuban	Yes, another Hispanic, Latino/a/x, or Spanish origin	Missing						
*N*	2437	251	51	17	144	130						
%	80.4	8.3	1.7	0.6	4.8	4.3						

### Procedure

The NCHAT study is a nationally representative, multi-method survey originally designed to focus on discrimination, racial trauma, health, psychological well-being, health behaviors, stressors, and time use. NCHAT recruited cohabiting and married individuals who were in a same- or different-gender relationship in the United States during the COVID- 19 pandemic (September 2020 to April 2021). The total sample was drawn from the Gallup Panel and the Gallup Recontact sample. Both sample sources are probability-based and representative of the U.S. adult population, and oversamples were drawn to ensure representation from minoritized communities (e.g., Black, Asian American, low-SES, LGBTQ + respondents). Participants were asked to complete a 40-min survey in either English or Spanish, after which they were asked to invite their partners to participate in NCHAT. Participants also completed a 24-h time-diary which was not included in this study. The current study only analyzed secondary survey data from the 1,515 couples who participated; there was no age restriction.

### Compliance with Ethical Standards

This study was performed in line with the principles of the Declaration of Helsinki. Approval was granted by the Northwestern University IRB for our use of NCHAT data (IRB No: STU00217911). Informed consent was obtained from all individual participants included in the study. The authors have no relevant financial or non-financial conflicts of interest to disclose.

### Materials

All measures for the present study originated from the main survey.

#### COVID Stress–Observed Indicators

From the larger survey, we selected 20 indicators of a latent factor of COVID stress. We specifically selected types of stress (and associated safety behaviors) that *most of the population* experienced during COVID, so that their differential impact on people identifying as SMs’ mental health could be validly compared. Our latent variable approach is helpful in this context because it only captures the variance that certain indicators (e.g., social distancing) share with others (e.g., stress about getting COVID), thus measuring the shared distress common to the following items. Participants rated the extent to which it was difficult to avoid COVID exposure at their work on a scale from 1 (strongly disagree) to 5 (strongly agree), and how worried they were about being exposed at work on a scale from 1 (not worried at all) to 4 (very worried). Next, an item assessed how much individuals were practicing social distancing (“Are you currently practicing social distancing as best you can, in other words: are you maintaining at least 6 feet of physical space between you and others to avoid spreading or catching the coronavirus?”) on a scale from 1 (not at all) to 5 (very much) in addition to an item (“To what extent has your life been affected or disrupted by the coronavirus situation?”) on a scale from 1 (not at all) to 4 (a great deal). Relevant items also assessed changes in stress due to COVID (“In the past week, have you been less stressed, more stressed, or had about the same amount of stress as before the coronavirus pandemic?”) on a scale from 1 (less stressed) to 3 (more stressed), and stress about 13 specific COVID-related situations (e.g., “getting coronavirus”) on a scale from 1 (not at all stressed) to 5 (very stressed). Individuals also rated changes in income due to COVID (“Was your household’s income in 2020 more than, less than, or about the same as your household's income in 2019?”) on a scale from 1 (less) to 3 (more), which was reverse coded. Lastly, participants answered a yes/no item about immunocompromised household members (“Do you personally have someone in your household who is likely to suffer serious complications if infected with the coronavirus?”). We intentionally did not include any indicators for COVID stress pertaining to relationship quality, caregiving, or childbearing to reduce the possibility of confounded partner effects.

#### Depression

Participants completed a 10-item version of the Center for Epidemiologic Studies Depression Scale (CES-D; Andresen et al., 1994; Radloff, 1977). Items (e.g., “I felt depressed”) were rated with respect to the past week on a scale from 1 (Rarely or none of the time) to 4 (most or all of the time). Relevant items were reverse scored, and all items were aggregated to create a total score where higher scores indicate more depression.

#### Anxiety

The Generalized Anxiety Disorder- 7 (GAD- 7; Spitzer et al., 2006) scale assessed participants’ anxiety experiences (e.g., “Feeling nervous, anxious or on edge”) regarding the past 7 days. Items were rated on a scale from 1 (Not at all) to 4 (Nearly every day) and aggregated to form a total score for which higher values indicate more anxiety.

#### Stress Overload

Participants completed the Short Stress Overload Scale (Amirkhan, 2018) regarding the past 7 days. Items (e.g., “Overwhelmed by your responsibilities”) are rated for frequency on a scale from 1 (never) to 5 (very often). Items were averaged to create a total score for which higher values indicate more stress.

#### Positive and Negative Coping

Respondents were asked “What are you doing to cope with the coronavirus pandemic?”, followed by a checklist of positive (e.g., praying or meditating, getting plenty of sleep) and negative (e.g., drinking alcohol, cutting or self-injury) coping behaviors. We counted the number of positive and negative coping behaviors for which higher values indicate more positive and/or negative coping (respectively).

#### Difficulties with Emotion Regulation

The Difficulties with Emotion Regulation Scale-Short Form (Kaufman et al., 2016) includes ten items assessing maladaptive emotion regulation (e.g., “When I am upset, it takes me a long time to feel better”). Participants reported frequency for each item on a scale from 1 (never) to 5 (very often). One item was reverse coded. All items were aggregated to form a total score where higher values indicate more difficulties with emotion regulation.

### Analyses

Before data were analyzed, correlation tables and descriptives were run in SPSS to rule out the possibility of nonnormality violating model assumptions or extreme multicollinearity between analytic variables. These analyses were unweighted.

#### Measurement Model

A measurement (i.e., factor) model was pursued to determine whether the proposed indicators of COVID stress load on a common factor; this approach is beneficial because latent factors do not include measurement error, and because we were initially unsure if the indicators were all iterations of a common construct. To parameterize this, a confirmatory factor analysis (CFA) was run in MPlus using full-information maximum likelihood/maximum-likelihood robust (FIML/MLR). This is ideal for this study, as it accounts for missing data without imputation (Enders & Bandalos, 2001; Muthén & Muthén, 2024). We entered raw data for all indicators simultaneously and specified the CLUSTER option to account for covariation between partners. Relevant variable(s) were declared as categorical. Fit was assessed via AIC and BIC because these are directly comparable metrics between non-nested models (Raftery, 1995). The STARTS option was used to aid in reaching an acceptable solution.^4^ CFAs were weighted using the WEIGHT_COUPLE variable.

#### Structural (Actor-Partner) Models

Next, we conducted actor-partner interdependence models (APIMs). These models fit direct effects from one’s own latent factor of COVID stress (estimation details above) to both one’s own *and* one’s partner’s outcome. To compare between people identifying as SMs and people identifying as non-SMs, the TYPE = MIXTURE COMPLEX and CLASSES and KNOWNCLASS option were used to run APIMs separately by self-reported sexual identity group (i.e., a multigroup model); although concordance on sexual identity is important, we were hesitant to assume partner knowledge of sexual identity, so we used only individual self-reports. Although sexual identity disclosure is measured in NCHAT, we believe that including information on sexuality outside of one’s own identity (e.g., disclosure within the partnership) would substantively change the research question and/or bias results given the dyadic focus. As with CFAs, the CLUSTER option was used for the couple ID and fit was evaluated using AIC and BIC (Raftery, 1995). Models were weighted using the WEIGHT_COUPLE variable.

**Fig. 1:**
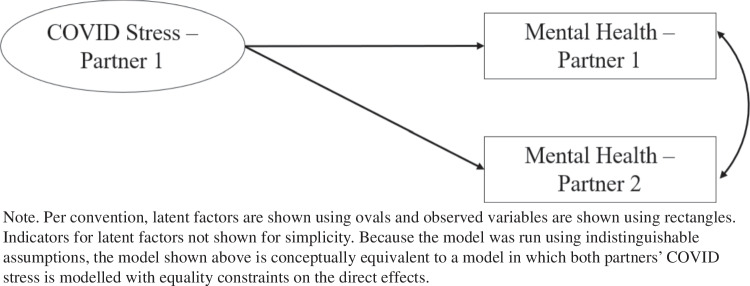
Conceptual diagram for latent variable APIMs. Note. Per convention, latent factors are shown using ovals and observed variables are shown using rectangles. Indicators for latent factors not shown for simplicity. Because the model was run using indistinguishable assumptions, the model shown above is conceptually equivalent to a model in which both partners’ COVID stress is modeled with equality constraints on the direct effects

Multigroup models were simultaneously estimated for people identifying as gay/lesbian, plurisexual,^5^ and straight, and for each outcome, totaling six multigroup APIMs. In all models, the latent factor of COVID stress was modeled only for the actor (i.e., the “index participant” who endorsed the sexual identity of that group), so there was only one actor effect and one partner effect. We also examined parameter equality between groups, which provided a statistical test of, for instance, whether COVID stress experienced by people identifying as straight affects their own and/or their partner’s mental health differently than COVID stress experienced by people identifying as gay/lesbian. All estimates are from the unstandardized solution.^6^ FIML/MLR estimation was used in all models, as well as ALGORITHM = INTEGRATION to aid in estimation (Fig. 1).

## Results

Descriptives for outcome variables are listed in Table 2, including N and skew/kurtosis. The confirmatory model (Table 3) had strong loadings from all indicators, although residual covariances were added between several indicators based on modification indices and substantive considerations.

**Table 2:** Descriptives for all outcome variables of individual NCHAT respondents

	*N*	M	SD	Skew	Kurtosis
Depression	2994	1.86	0.62	0.82	0.09
Anxiety	2994	1.74	0.74	1.15	0.69
Stress	2994	2.38	0.96	0.63	− 0.20
Difficulties with emotion regulation	2993	2.41	0.86	0.46	− 0.30
Count of positive coping behaviors	2990	3.97	1.70	0.03	− 0.35
Count of negative coping behaviors	2990	1.15	1.02	0.80	0.33

**Table 3:**
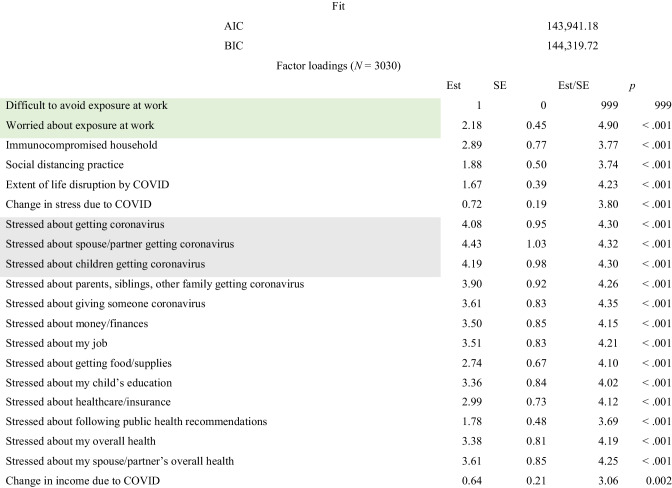
Fit and loadings for confirmatory factor model of COVID stress of individual NCHAT respondents

Note. Gray highlighting denotes indicators with residual covariances added

### Multigroup Actor-Partner Interdependence Models

#### Depression

In all three groups, experiencing more COVID stress tended to associate with higher depression for oneself (Table 4; Actor Est Range = 1.25–1.40, *P*s < 0.01) and one’s partner (Partner Est Range = 0.58–0.87, *P*s < 0.05). In the groups for people identifying as gay/lesbian (Est = 0.05, *P* < 0.01) and straight (Est = 0.06, *P* < 0.001), outcome covariances suggested that partners tended to be similar on their level of depression.

**Table 4:** Results and multigroup comparisons for latent variable APIMs

	Est	SE	Est/SE	*p*		Est	SE	Est/SE	*p*
**Depression model**									
Group straight (*N* = 1767)					Comparing actor effects				
Actor effect	**1.25**	**0.27**	**4.61**	** <.001**	Straight vs gay/lesbian	− 0.13	0.17	− 0.78	0.44
Partner effect	**0.58**	**0.15**	**3.86**	** <.001**	Straight vs plurisexual	− 0.15	0.32	− 0.47	0.64
Outcome covariance	**0.06**	**0.02**	**4.37**	** <.001**	Gay/lesbian vs plurisexual	− 0.02	0.34	− 0.07	0.95
Group gay/lesbian (*N* = 782)					Comparing partner effects				
Actor effect	**1.38**	**0.33**	**4.22**	** <.001**	Straight vs gay/lesbian	− 0.09	0.17	− 0.55	0.59
Partner effect	**0.68**	**0.20**	**3.38**	**0.001**	Straight vs plurisexual	− 0.29	0.32	− 0.89	0.38
Outcome covariance	**0.05**	**0.02**	**2.72**	**0.007**	Gay/lesbian vs plurisexual	− 0.19	0.34	− 0.56	0.57
Group plurisexual (*N* = 412)					Comparing outcome covariances				
Actor effect	**1.40**	**0.47**	**2.98**	**0.003**	Straight vs gay/lesbian	0.01	0.02	0.46	0.65
Partner effect	**0.87**	**0.36**	**2.43**	**0.02**	Straight vs plurisexual	0.06	0.04	1.69	0.09
Outcome covariance	0.002	0.04	0.06	0.96	Gay/lesbian vs plurisexual	0.05	0.04	1.27	0.21
**Anxiety model**									
Group straight (*N* = 1767)					Comparing actor effects				
Actor effect	**1.40**	**0.30**	**4.66**	** <.001**	Straight vs gay/lesbian	− 0.17	0.21	− 0.83	0.40
Partner effect	**0.58**	**0.16**	**3.67**	** <.001**	Straight vs plurisexual	− **0.82**	**0.37**	− **2.24**	**0.03**
Outcome covariance	**0.07**	**0.02**	**2.99**	**0.003**	Gay/lesbian vs plurisexual	− 0.65	0.39	− 1.67	0.10
Group gay/lesbian (*N* = 782)					Comparing partner effects				
Actor effect	**1.57**	**0.35**	**4.46**	** <.001**	Straight vs gay/lesbian	− 0.11	0.25	− 0.46	0.65
Partner effect	**0.69**	**0.25**	**2.80**	**0.005**	Straight vs plurisexual	− 0.54	0.30	− 1.78	0.08
Outcome covariance	0.04	0.04	1.18	0.24	Gay/lesbian vs plurisexual	− 0.43	0.37	− 1.14	0.25
Group plurisexual (*N* = 412)					Comparing outcome covariances				
Actor effect	**2.22**	**0.58**	**3.84**	** <.001**	Straight vs gay/lesbian	0.02	0.04	0.58	0.56
Partner effect	**1.12**	**0.36**	**3.12**	**0.002**	Straight vs plurisexual	− 0.01	0.06	− 0.07	0.94
Outcome covariance	0.07	0.06	1.14	0.25	Gay/lesbian vs plurisexual	− 0.03	0.07	− 0.40	0.69
**Stress model**									
Group straight (*N* = 1767)					Comparing actor effects				
Actor effect	**1.79**	**0.39**	**4.53**	** <.001**	Straight vs gay/lesbian	− 0.30	0.22	− 1.39	0.17
Partner effect	**0.70**	**0.20**	**3.51**	** <.001**	Straight vs plurisexual	− 0.86	0.45	− 1.93	0.05
Outcome covariance	**0.14**	**0.03**	**4.23**	** <.001**	Gay/lesbian vs plurisexual	− 0.56	0.43	− 1.32	0.19
Group gay/lesbian (*N* = 782)					Comparing partner effects				
Actor effect	**2.09**	**0.47**	**4.43**	** <.001**	Straight vs gay/lesbian	− 0.44	0.32	− 1.39	0.17
Partner effect	**1.14**	**0.38**	**2.97**	**0.003**	Straight vs plurisexual	− **1.12**	**0.49**	− **2.27**	**0.02**
Outcome covariance	0.05	0.06	0.96	0.34	Gay/lesbian vs plurisexual	− 0.68	0.48	− 1.42	0.16
Group plurisexual (*N* = 412)					Comparing outcome covariances				
Actor effect	**2.65**	**0.72**	**3.68**	** <.001**	Straight vs gay/lesbian	0.09	0.07	1.32	0.19
Partner effect	**1.82**	**0.58**	**3.17**	**0.002**	Straight vs plurisexual	− 0.02	0.10	− 0.22	0.83
Outcome covariance	0.16	0.09	1.72	0.09	Gay/lesbian vs plurisexual	− 0.11	0.11	− 0.98	0.33
**Difficulties with emotion regulation model**									
Group straight (*N* = 1767)					Comparing actor effects				
Actor effect	**1.55**	**0.34**	**4.49**	** <.001**	Straight vs gay/lesbian	− 0.27	0.21	− 1.32	0.19
Partner effect	**0.54**	**0.17**	**3.22**	**0.001**	Straight vs plurisexual	− **0.90**	**0.41**	− **2.17**	**0.03**
Outcome covariance	**0.08**	**0.03**	**3.14**	**0.002**	Gay/lesbian vs plurisexual	− 0.63	0.41	− 1.52	0.13
Group gay/lesbian (*N* = 782)					Comparing partner effects				
Actor effect	**1.82**	**0.41**	**4.44**	** <.001**	Straight vs gay/lesbian	− 0.50	0.31	− 1.62	0.11
Partner effect	**1.04**	**0.35**	**3.00**	**0.003**	Straight vs plurisexual	− 0.57	0.48	− 1.17	0.24
Outcome covariance	**0.13**	**0.04**	**3.17**	**0.002**	Gay/lesbian vs plurisexual	− 0.06	0.51	− 0.13	0.90
Group plurisexual (*N* = 412)					Comparing outcome covariances				
Actor effect	**2.45**	**0.65**	**3.79**	** <.001**	Straight vs gay/lesbian	− 0.05	0.05	− 1.10	0.27
Partner effect	**1.10**	**0.51**	**2.15**	**0.03**	Straight vs plurisexual	0.08	0.09	0.91	0.36
Outcome covariance	− 0.001	0.09	− 0.02	0.99	Gay/lesbian vs plurisexual	0.13	0.10	1.37	0.17
**Negative coping model**									
Group straight (*N* = 1767)					Comparing actor effects				
Actor effect	**1.15**	**0.27**	**4.28**	** <.001**	Straight vs gay/lesbian	− 0.22	0.27	− 0.82	0.41
Partner effect	**0.80**	**0.21**	**3.72**	** <.001**	Straight vs plurisexual	− **1.21**	**0.58**	− **2.09**	**0.04**
Outcome covariance	**0.33**	**0.06**	**5.85**	** <.001**	Gay/lesbian vs plurisexual	− 0.99	0.63	− 1.57	0.12
Group gay/lesbian (*N* = 782)					Comparing partner effects				
Actor effect	**1.37**	**0.35**	**3.89**	** <.001**	Straight vs gay/lesbian	− 0.09	0.27	− 0.33	0.74
Partner effect	**0.89**	**0.29**	**3.02**	**0.003**	Straight vs plurisexual	− 0.05	0.36	− 0.15	0.88
Outcome covariance	**0.27**	**0.08**	**3.26**	**0.001**	Gay/lesbian vs plurisexual	0.04	0.40	0.09	0.93
Group plurisexual (*N* = 412)					Comparing outcome covariances				
Actor effect	**2.36**	**0.66**	**3.58**	** <.001**	Straight vs gay/lesbian	0.05	0.09	0.56	0.57
Partner effect	**0.85**	**0.39**	**2.18**	**0.03**	Straight vs plurisexual	**0.33**	**0.14**	**2.45**	**0.01**
Outcome covariance	− 0.01	0.13	− 0.04	0.97	Gay/lesbian vs plurisexual	0.28	0.16	1.77	0.08
**Positive coping model**									
Group straight (*N* = 1767)					Comparing actor effects				
Actor effect	− 0.46	0.27	− 1.74	0.08	Straight vs gay/lesbian	0.39	0.63	0.61	0.54
Partner effect	− **0.77**	**0.30**	− **2.61**	**0.009**	Straight vs plurisexual	− 0.57	0.55	− 1.04	0.30
Outcome covariance	**0.71**	**0.12**	**5.92**	** <.001**	Gay/lesbian vs plurisexual	− 0.95	0.76	− 1.26	0.21
Group gay/lesbian (*N* = 782)					Comparing partner effects				
Actor effect	− 0.85	0.61	− 1.40	0.16	Straight vs gay/lesbian	0.32	0.48	0.66	0.51
Partner effect	− **1.09**	**0.45**	− **2.40**	**0.02**	Straight vs plurisexual	− 1.21	0.65	− 1.85	0.06
Outcome covariance	0.43	0.22	1.94	0.05	Gay/lesbian vs plurisexual	− **1.53**	**0.74**	− **2.07**	**0.04**
Group plurisexual (*N* = 412)					Comparing outcome covariances				
Actor effect	0.11	0.50	0.21	0.83	Straight vs gay/lesbian	0.28	0.24	1.14	0.26
Partner effect	0.44	0.57	0.76	0.45	Straight vs plurisexual	0.05	0.36	0.14	0.89
Outcome covariance	0.66	0.36	1.83	0.07	Gay/lesbian vs plurisexual	− 0.23	0.41	− 0.56	0.58

Note: Significant effects and comparisons are bolded

Actor/partner effects did not significantly differ between groups, and there were no group differences in the outcome covariances.

#### Anxiety

In all three groups, experiencing more COVID stress tended to associate with higher anxiety for oneself (Actor Est Range = 1.40–2.22, *P*s < 0.001) and one’s partner (Partner Est Range = 0.58–1.12, *P*s < 0.01). In the group of individuals identifying as straight, outcome covariances suggested that individuals’ anxiety tended to associate with their partners’ anxiety (Est = 0.07, *P* < 0.01).

Group differences suggested that COVID stress for people identifying as plurisexual impacted their own anxiety more strongly than COVID stress did for people identifying as straight (Est = − 0.82, *P* = 0.025).

#### General Stress

Actor (Est Range = 1.79–2.65, *P*s < 0.001) and partner (Est Range = 0.70–1.82, *P*s < 0.01) effects were significant in all groups, suggesting that one’s own COVID stress displays associations with one’s own, and one’s partner’s, level of general stress. Outcome covariances were also significant, but only for the group identifying as straight (Est = 0.14, *P* < 0.001).

COVID stress for people identifying as plurisexual tended to impact their partners’ general stress more strongly compared to people identifying as straight (Straight vs Plurisexual Est = − 1.12, *P* = 0.023).

#### Difficulties with Emotion Regulation

Actor and partner effects were significant for all three groups, suggesting that one’s own COVID stress had direct associations with one’s own (Est Range = 1.55–2.45, *P*s < 0.001) and one’s partner’s (Est Range = 0.54–1.10, *P*s < 0.05) emotion regulation regardless of whether people identified as SM. There were also significant outcome covariances in groups identifying as straight (Est = 0.08, *P* < 0.01) and gay/lesbian (Est = 0.13, *P* < 0.01). In terms of between group differences, COVID stress for people identifying as plurisexual impacted their own emotion regulation more strongly than COVID stress did for people identifying as straight (Est = − 0.90, *P* = 0.030).^7^

#### Negative Coping

Actor/partner effects were significant for all three groups, suggesting that higher COVID stress tends to correspond with more negative coping behaviors for oneself (Est Range = 1.15–2.36, *Ps* < 0.001) and one’s partner (Est Range = 0.80–0.89, *Ps* < 0.05). In addition, groups of individuals identifying as straight and gay/lesbian reported that they were relatively concordant with their partners on the number of negative coping techniques they used (Est Range = 0.27–0.33, *P*s < 0.01). There were two significant group differences. First, people identifying as straight tended to be more concordant with their partners on number of negative coping strategies they employed compared to the group of people identifying as plurisexual (Est = 0.33, *P* = 0.014). Second, COVID stress for people identifying as plurisexual impacted their own negative coping more strongly than COVID stress did for people identifying as straight (Est = − 1.21, *P* = 0.037).

#### Positive Coping

Partner effects in the groups of people identifying as straight (Est = − 0.77, *P* < 0.01) and gay/lesbian (Est = − 1.09, *P* < 0.05) suggested that the more COVID stress these individuals experienced, the fewer positive coping strategies their *partners* used. Significant outcome covariances in the group of people identifying as straight (Est = 0.71, *P* < 0.001) suggested that these individuals and their partners tended to report being concordant on their number of positive coping strategies. Lastly, COVID stress for people identifying as plurisexual had a nonsignificant *positive* influence on their partners’ positive coping (Est = 0.44, *P* = 0.445) compared to people identifying as gay/lesbian, whose COVID stress exhibited a significant *negative* influence on their partners’ positive coping (Est = − 1.09, *P* = 0.016; Contrast Est = − 1.53, *P* = 0.038).

## Discussion

The disproportionate impact of COVID- 19 pandemic stress for *individuals* identifying as SMs (Abramovich et al., 2022; Dyar et al., 2022; Fallahi et al., 2022; Gutman et al., 2022) necessitates examining whether this stress disproportionately exacerbated mental health inequities for *couples*. Using a dyadic dataset from the National Couples’ Health and Time (NCHAT) study, we tested the differential impacts of latent COVID- 19 stress on mental health among people identifying as straight, gay/lesbian, and plurisexual and their partners. This dyadic approach is an innovation on prior work and explicitly models a primary context of stress proliferation and coping during the COVID- 19 pandemic (i.e., romantic partnerships).

For all groups, COVID stress had significant and detrimental impacts on depression, anxiety, stress, emotion regulation, and negative coping strategies, with few significant differences in actor/partner effects. This is partially consistent with expectations, and with prior work showing that people identifying as SMs have relationships that function similarly to the relationships of people identifying as non-SMs (Kurdek, 1992, 2004). This is also supported by work showing that the COVID- 19 pandemic exercised nearly universal impacts on population health, including mental health (Mullin et al., 2022). This result partially contrasts, however, with research showing that people identifying as SMs experienced more COVID stress (Abramovich et al., 2022; Kamp Dush et al., 2022; Oren, 2022; Salerno et al., 2022) and pre-pandemic mental health concerns (Wittgens et al., 2022). Taken together, it is possible that SM couples’ shared support—developed in the face of longstanding societal oppression (Diamond & Alley, 2022; Domínguez et al., 2015)—resulted in a better coping response to the COVID- 19 pandemic, and similar associations of COVID stress with mental health relative to people identifying as non-SMs.

Although the *overall* pattern of results suggested few actor/partner differences between groups, several important nuances emerge when examining *individual* findings. People identifying as plurisexuals tended to display stronger actor effects of COVID stress on anxiety, emotion regulation, and negative coping, as well as stronger partner effects on general stress, compared to people identifying as straight and people identifying as gay/lesbian. The group identifying as plurisexual also tended to be less concordant with partners in terms of their mental health. Lastly, individuals identifying as plurisexual exhibited a partner effect in the opposite direction compared to individuals identifying as gay/lesbian. Taken together, these results could suggest (as shown in prior studies) that people identifying as plurisexual experience wider disparities in mental health, even compared to other people identifying as SMs (Feinstein & Dyar, 2017; Feinstein et al., 2021). Although these findings do not speak to whether people identifying as SMs experienced more COVID-related stress (which has been well-documented in prior work and was therefore not our main focus; Goldey et al., 2022; Oren, 2022), they do show that *the same amount* of COVID stress will have a larger (and sometimes opposite-direction) impact on people identifying as plurisexual—and their partners’—mental health compared to other groups. It is possible that people identifying as plurisexual lacked a “true” community upon which to lean during the pandemic (i.e., since people identifying as plurisexual experience “double discrimination” from people identifying as SMs and people identifying as non-SMs; Ochs, 1996). Under a condition of severe societal stress (i.e., COVID), this may have been especially likely to occur and may have detrimented the mental health of people identifying as plurisexual even further. Alternatively, prior research shows that similarity to a partner, even on presumably “negative” constructs (e.g., substance use, personality pathology; Homish & Leonard, 2007; Smith et al., 2020), is associated with better relationship functioning. Since the mental health of people identifying as plurisexual was more dissimilar to their partners’ in this study, it is possible that—in addition to the basic effects of COVID stress—they did not have a dyadic “safe space” to turn to wherein their challenges would be understood and validated. Regardless, these findings are preliminary and should be replicated. In particular, it may behoove future researchers to investigate these questions using qualitative methods.

### Policy Implications

This paper shows that—while SM individuals experience more stress—this stress is similarly associated with mental health for SM-identified vs. non-SM couples, with some notable differences for SMs identifying as plurisexual. Although it’s not necessarily positive that SM couples have needed to develop such extreme coping skills, this has broader implications for responding to other large, global events that may occur in the future. Specifically, close relationships could be leveraged as powerful tools to improve individual health (e.g., partners being encouraged through policy or public messaging to assist one another in completing individual self-care; Kauffman & Silberman, 2009) for all portions of the population. For instance, broad public health messaging may consider incorporating themes related to the intentional and repeated reliance on close others (e.g., partners, former partners, friends; Hull & Ortyl, 2019; Jacmin-Park et al., 2022; Kalb et al., 2020; Quinn et al., 2022), and public policy could specifically center the needs of marginalized—over dominant—communities.

### Limitations

Several limitations should be acknowledged. First, all data were cross-sectional, and other sources of information contained in NCHAT (e.g., the time diary) may be more illustrative for within-person associations. We also did not incorporate whether partners identified as SMs. Although this was necessary based on the structure of the data (i.e., partners were not necessarily aware of each other’s sexual orientations/identities, differing abilities to distinguish dyad members, differing SM concordance), it will be important for future work to consider dyad-level indicators of vulnerability to inequity. Lastly, it is possible that other factors (e.g., experiences of racial trauma during COVID) could have shaped the associations we observed and are important to incorporate in future work.

## Conclusions

We examined whether—and how—COVID-related stress exercised a disproportionate impact on the mental health of people identifying as SMs and their partners. Although results suggested widespread impacts of COVID stress on mental health across sexual orientation/identity, people identifying as plurisexuals tended to experience the strongest impacts and were the least concordant with their partners. These results, if replicated, may suggest avenues for future prevention and public health messaging.

## Supplementary Information

Below is the link to the electronic supplementary material.

Supplementary file1 (XLSX 35 KB)

## Data Availability

Data for the present study are publicly available through ICPSR.

## Footnotes

1 Throughout this manuscript, when referring to COVID, COVID- 19, and the COVID- 19 pandemic, we mean the societal response and management of the ongoing pandemic, rather than SARS-CoV- 2 itself.

2 We originally aimed to run analyses separately by gender identity (see analytic details below). However, because of the severe imbalance in gender identity, this was not feasible and thus not attempted for the present report.

3 Due to restrictions on use of NCHAT data, certain demographic characteristics (e.g., Min/Max values) cannot be reported to protect anonymity and avoid incidental disclosure.

4 The CFA was re-run including covariates of age (Est =.000; *p* =.896), gender identity (cisgender/noncisgender; Est =.151, *p* =.008), and sexual orientation/identity (heterosexual/sexual minority; Est =.121, *p* =.001) on the latent factor. Full results for this model can be viewed in supplement.

5 The group for individuals identifying as plurisexual was not disaggregated by gender of the partner, because few individuals (*N* = 74) were partnered with people identifying as straight, while the rest identified as gay/lesbian or plurisexual (i.e., included in the *N* = 412). Because the cell sizes would be small, and because of NCHAT reporting rules designed to prevent deductive disclosure, we do not believe we would be able to report these results. In addition, there were concerns regarding power due to these small cell sizes that would also preclude the analysis.

6 All actor-partner models were re-run with age and gender identity (cisgender/noncisgender) as covariates on the latent factor of COVID stress, and on the actor/partner outcomes. Results were largely unchanged, and models including covariates are presented in supplement.

7 This was the only comparison to change upon the inclusion of covariates; in the model including age and gender identity, it fell below significance (Est = −.76, *P* =.051).

## Code Availability

Available on request from the corresponding author.
